# TCP Doped with Metal Ions Reinforced with Tetragonal and Cubic Zirconia

**DOI:** 10.3390/biomimetics8080599

**Published:** 2023-12-12

**Authors:** Vanessa M. Ferro, Beatriz C. Silva, Duarte F. Macedo, Natanael F. Fernandes, Abílio P. Silva

**Affiliations:** 1 C-MAST—Centre for Mechanical and Aerospace Science and Technologies, Universidade da Beira Interior, Rua Marquês d’Ávila e Bolama, 6201-001 Covilhã, Portugal; vanessa.ferro@ubi.pt (V.M.F.);; 2 CICS-UBI—Centro de Investigação em Ciências da Saúde, Universidade da Beira Interior, Av. Infante D. Henrique, 6200-506 Covilhã, Portugal

**Keywords:** bioceramic composites, TCP, ZrO_2_, metal ions, bone regeneration, biocompatibility

## Abstract

Ceramic biocomposites based on bioactive tricalcium phosphate doped with metal ions are a strategy for obtaining good biomimetics for human bone composition. Manufacturing with PMMA porogen also induces bone-like porosity morphology. The poor strength of tricalcium phosphate can be overcomed by designing ceramic composites reinforced with tetragonal and cubic zirconia. In this work, five different bioceramic composites were manufactured without and with induced porosity and their physical, mechanical, microstructural, and biological properties were studied. With the addition of tetragonal and cubic zirconia, an improvement in strength of 22% and 55%, respectively, was obtained, corresponding to up to 20.7 MPa. PMMA was suitable for adding porosity, up to 30%, with interconnectivity while an excellent hOB cellular viability was achieved for all biocomposites.

## 1. Introduction

The average life expectancy has increased, causing the aging of the world population. With this, health complications related to joints and bone tissue, such as osteoporosis and osteoarthritis, can arise [1,2]. Therefore, finding alternatives that provide quality of life to the patient is essential. Inert bioceramics (e.g., zirconia, ZrO_2_), do not form biochemical bonds with surrounding tissues, i.e., do not react with the body [3]. Physical, chemical, and mechanical properties such as high compressive strength, wear and corrosion resistance, hardness, elastic modulus similar to that of steel, high fracture toughness, and stability in a physiological environment, make ZrO_2_ a material of the highest interest for the manufacture of orthopedic and dental prostheses [2,4].

At room temperature and in pure form, ZrO_2_ has a stable monoclinic crystalline structure (m-ZrO_2_) up to 1170 °C. With increasing temperature, it becomes metastable: tetragonal (t-ZrO_2_) stable up to 2370 °C and above this temperature, cubic (c-ZrO_2_) [4,5,6]. The m-ZrO_2_ is not suitable for high temperature applications due to the volume expansion associated with the transformation of t-ZrO_2_ to m-ZrO_2_ [7,8], known as martensitic transformation [5,9]. In this transformation there is an increase in volume of approximately 4.5% during cooling [6,8] which is detrimental to the mechanical behavior of ZrO_2_ because the stress induced during this transformation leads to the formation of cracks [5]. In this sense, it can be concluded that the oral environment is a strongly predisposing factor for uncontrolled martensitic transformation [6].

Stabilizers such as yttrium oxide (Y_2_O_3_) can be added to ZrO_2_ to inhibit the transformation of the t-phase to m-phase. Briefly, this addition results in the part of the Zr^+4^ atoms being replaced by Y^+3^ atoms, stabilizing the polymorphic modifications of ZrO_2_ when subjected to the sintering process. This avoids the volume variations caused by phase transformations [6,10] that form yttrium-stabilized zirconia (YSZ), where 3YSZ is mostly tetragonal and 8YSZ is mostly the cubic crystalline phase [11]. However, several studies describe that the use of Y_2_O_3_ has disadvantages mainly in its degradation at low temperature; this phenomenon is known as “Aging Phenomenon” while its use is limited by unpredictable behavior in the body [1,6,9,12].

On the other hand, active bioceramics can establish connections with the surrounding tissue and suffer progressive degradation as the new tissues form [13]. TCP can be used in cement and bone implants for its bioactivity, osteoinduction, and bioresorbability [14,15]. By its reabsorbable nature, part of the material dissolves, providing raw material for the growth and maintenance of the new tissue, facilitating osteoconduction [13,16]. Chemically, it is Ca_3_(PO_4_)_2_ and characterized by a Ca/P ratio 1.5 [17]. As a function of the temperature at which it is sintering, it can present three polymorphic forms: β-TCP (~<1150 °C), α-TCP (~1150–1460 °C), and α’-TCP (~>1460 °C) [18,19].

Metal ions such as magnesium (Mg^2+^), manganese (Mn^2+^), zinc (Zn^2+^), iron (Fe^3+^), and strontium (Sr^2+^), among others, can be added to form biomimetic bone composition and also improve several properties of these bioceramics [14,20]. Considering the cations in the human body, magnesium is the fourth in abundance and the second intracellular cation in tissues [21,22]. The human body contains about 30 g of magnesium, 50% stored in bones [23]. Magnesium plays a vital role in many enzymatic reactions, among which are the transmission of nerve impulses and the synthesis of fatty acids and proteins. Also, Mg^2+^ has an elemental role in biology because ATP needs to be bound to a magnesium ion to be biologically active as well as in the formation of the transition state where ATP is synthesized from ADP and inorganic phosphate. Mg^2+^ ions can replace Ca^2+^ ions by increasing the transformation temperature of β to α, stabilizing β-TCP, increasing mechanical strength, and improving osteoblast function and biocompatibility. Moreover, Mn^2+^ ions improve mechanical properties and corrosion resistance, while Zn^2+^ improves osteoblast function, corrosion resistance, and has antibacterial action. Other cations, such as Fe^3+^ preserve the structural stability of bioceramics and increase osseointegration, while Sr^2+^ inhibits bone resorption and stimulates the proliferation of osteoblasts and bone formation [14,20,24,25,26,27,28,29].

However, besides the essential effect of metal ions for human life, higher doses can be toxic [23]. For instance, levels of Mg^2+^ above 1.1 mM are generally considered hypermagnesemic. This effect promotes patients clinically to suffer from nausea, vomiting, lethargy, headaches, and/or flushing. When Mg^2+^ levels rise above 3.0 mM, it can cause serious heart defects, characterized by brachycardia, hypotension, and in extreme hypermagnesemia can result in coma, asystole, and death from cardiac arrest [22].

Manganese (Mn^2+^) ions accumulate in the bones, liver, pancreas and mainly in the brain. Pathologies, as polycythemia, dystonia, hepatic cirrhosis, have been related to excess of manganese. In particular, symptoms as in Parkinsonism have been reported to overexposure of manganese. The molecular mechanisms involved include oxidative stress, protein misfolding, apoptosis, mitochondrial dysfunctions, and interference in the homeostasis of other metal essential ions. The Mn^2+^ amount should not exceed 5 mg/m^3^, even for short periods; however, its toxicity depends on its chemical form: -Mn^2+^, the common form, is not dangerous but MnO_4_^−^ is very toxic [23].

High concentration of Zinc (Zn^2+^) can be found in vesicles in the brain, in bones, and in muscles. It is reported that the recommended dose is around 15 mg/day [23]. Poisoning with zinc phosphide, a rodenticide, causes cardiovascular, respiratory, renal, and hepatobiliary failure, among other complications [30].

In vitro studies have shown that high doses of iron could lead to osteoblast apoptosis via caspase 3. Iron overload also decreases the formation of mineralization nodes and inhibits growth of hydroxyapatite crystals, altering their crystallinity [30]. The toxicity generated by the excess of iron can cause cirrhosis, liver carcinoma, heart failure, diabetes mellitus, and osteoporosis. Therefore, in the presence of molecular oxygen, loosely bound iron is able to undergo a redox cycle (Fe^3+^/Fe^2+^) generating poisonous oxygen-derived free radicals [23].

For successful applications, bone structures must mimic the porosity of native bone and allow it to grow through the interconnectivity of the structure [31]. Polymethylmethacrylate (PMMA) has been considered for its particular characteristics, such as mechanical strength, moldability to fill complex defects, low cost, having approval by the FDA and with clean and easy thermal elimination at high temperatures [32,33,34].

The aim of this work was to produce dense ceramic biocomposites and PMMA induced porous ceramic biocomposites, both compositions based in TCP, doped with metal ions of magnesium (Mg^2+^), manganese (Mn^2+^), zinc (Zn^2+^), and iron (Fe^3+^). The two types of manufactured samples were reinforced with 10 wt% and 20 wt% of tetragonal zirconia (3YSZ) and cubic zirconia (8YSZ). A detailed evaluation of their physical, mechanical, microstructural, and biological properties was performed.

## 2. Materials and Methods

### 2.1. Materials and Manufacture Methods

Following the procedure described in the literature [14], TCP was doped with 10 mol% of Mg^2+^ and 5 mol% of a mixture of Zn^2+^, Mn^2+^, and Fe^3+^, totaling 15 mol% of metal ions. Being similar to the maximum amount of Ca^2+^ ions that can be replaced in the TCP structure, substituted TCP (sTCP) was obtained in this way [24]. For this purpose, calcium carbonate (CaCO_3_, PanReac AppliChem, Barcelona, Spain), ammonium phosphate dibasic ((NH_4_)_3_HPO_4_ Acros organics, Geel, Belgium, 98+%, CAS: 423375000), magnesium oxide (MgO, Alfa Aesar, Ward Hill, MA, USA, 96% min, CAS: 1309-48-4), manganese (II) oxide (MnO, 99%, Alfa Aesar, Ward Hill, MA, USA, CAS 011870-36), iron (III) oxide (Fe_2_O_3_, 99.9%, Alfa Aesar, Ward Hill, MA, USA, CAS: 1309-37-1), and zinc oxide (ZnO, 99.0%, PanReac AppliChem, Barcelona, Spain, CAS: 1314-13-2) were used. Also, ZrO_2_ stabilized with 3 mol% of yttrium (3YSZ, TZ-3Y, t-ZrO_2_, Tosoh, Tokyo, Japan, Lot: Z308782P), ZrO_2_ stabilized with 8 mol% of yttrium (8YSZ, TZ-8Y, c-ZrO_2_, Tosoh, Tokyo, Japan, Lot: Z807724P), and PMMA microspheres (Acros Organics, Geel, Belgium, CAS: 9011-14-7), with average size of 100 µm, were also added to induce porosity in some samples. Calcium carbonate, ammonium phosphate dibasic, 10 mol% of Mg, and 1.67 mol% of Zn^2+^, 1.67 mol% of Mn^2+^ and 1.67 mol% of Fe^3+^ in total amount of 50 g was mixed with 100 g of isopropyl alcohol (Labchem, Laborspirit, Santo Antão do Tojal, Portugal) in a high energy ball mill (Fritsch, Pulverisette 6, Idar-Oberstein, Germany) at 500 rpm for 150 min, divided into cycles of 30 min. To promote this mixing and simultaneous milling process, 50 g of YSZ spheres (Fritsch, Idar-Oberstein, Germany) were used. The mixture was dried in a stove (Carbolite, NR200-F, Derbyshire, UK) at 60 °C for 48 h. After this process, the mixture was sieved (Retsch, AS200, Düsseldorf, Germany) up to 63 μm to remove the YSZ spheres and some agglomerates. The laser diffraction method (Beckman Coulter, LS200, Brea, CA, USA) was used to analyzed the particle size distribution of the powders after milling. Then, the powder was placed in crucibles and calcined in an electric furnace (Termolab, MLR, Águeda, Portugal) in air for 10 h at a temperature of 1000 °C. After calcination of the sTCP reference material, the other four ceramic composites with 3YSZ and 8YSZ were mixed according to the compositions and nomenclatures summarized in Table 1.

**Figure 1:**
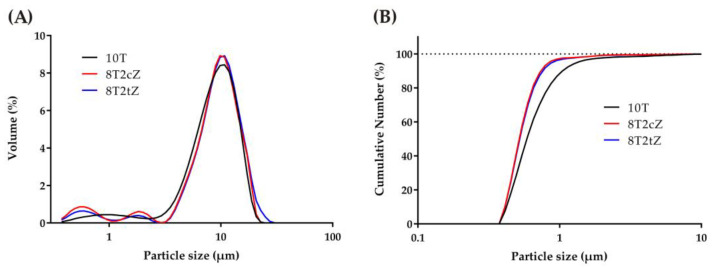
Particle size distribution of powders of the mixtures 10T (sTCP), 8T2cZ, and 8T2tZ: (**A**) differential volume; (**B**) cumulative number.

To each of the compositions with YSZ, 3YSZ and 8YSZ were added according to the corresponding mass fraction. Then, 15 g of the doped or substituted TCP (sTCP) was added according to the mass fraction, and 15 g of YSZ balls and 30 g of isopropyl alcohol were mixed in a high energy ball mill [35]. The milling, drying, and sieving processes occurred under the previous conditions. The particle size distribution (PSD) of the powders of the reference material (sTCP, which give rise to the material designated 10T) and the other compositions with the mixture between 80% of sTCP, 20% of cubic zirconia (8T2cZ), and 20% of tetragonal zirconia (8T2tZ) are shown in Figure 1.

The PSD of 10T powders is bimodal: with a major peak at 10 µm and another minor peak at 1 µm. Both compositions have zirconia (8T2cZ and 8T2tZ) present, instead of one, with two small minor peaks of 0.5 and 1.8 microns, respectively.

To induce porosity, PMMA spheres were added to each of the five compositions in the mass fraction 60:40 (each of the five compositions: PMMA, respectively). In order to obtain a homogeneous mixture, the mixture was placed in the Fritsch mill for 20 s at 500 rpm.

Cylindric pellets of bioceramic composites with mass of ~0.65 g, ~13 mm diameter and 3 mm thickness were made using a universal electromechanical testing machine (Shimadzu, AGS-X, Kyoto, Japan) with a 13 mm diameter stainless steel matrix in which uniaxial pressure of 50 MPa was applied for 10 s. Then, the samples were sintered in air at a temperature of 1300 °C for 120 min with a heating rate of 5 °C/min.

### 2.2. Microstructural and Mechanical Characterization

To confirm if the doping reaction occurred, the XRD test was performed in the dense biocomposites 10T, 8T2tZ, and 8T2cZ, since 8T2tZ and 8T2cZ have the highest concentration of each ZrO_2_ studied and 10T the same concentration of each ion without the addition of 3YSZ and 8YSZ. An X-ray diffractometer (DMAX III/C, Rigaku, Tokyo, Japan) with the Bragg-Brentano (θ/2θ) horizontal geometry was used. The X-ray tube of copper (wavelength of 1.5405 Å) operated at 40 kV at 30 mA using CuKα radiation. The intensity of diffracted radiation as function of the 2θ diffraction angle was obtained between 5° and 90°. The diffractograms obtained were compared with the theoretical cards available in the ICDD database of the MDI/JADE, version 6 analysis software. The contents of each crystalline phase of the compositions in %vol were quantified through Rietveld refinements using the FullProf software, version May2021 [36]. Scanning electron microscopy (SEM) (Hitachi S-2700, Tokyo, Japan), was performed for microstructure imaging by applying the SE mode at an accelerating voltage of 20 kV. Chemical analysis was performed using SEM with energy dispersive X-ray probe (EDX, Brucker Quantax 400, Elk Grove Village, IL, USA). This analysis was carried out using the average of three different areas, where the gold peaks were not considered and all other peaks corresponding to Ca, P, and O and also the metals such as Zr, Mg, Mn, Fe, and Zn were quantified.

Fourier transform infrared spectroscopy (FTIR) was performed using a Nicolet^TM^ iS 10 FTIR spectrometer (Thermo Scientific Inc., Waltham, MA, USA). Infrared spectra were recorded in the range of 525–4000 cm^−1^ at ambient temperature and with a resolution of 4 cm^−1^ (32 scans).

Apparent porosity and bulk density were measured according to ASTM C20-00 [37]. In this procedure three different weights, in grams, were calculated: dry sample weight (D), saturated weight (W), after boiling for 2 h and resting 12 h entirely covered with water, and suspended immersed in water weight (S). Considering the density of the water equal to 1 g/cm^3^, the apparent porosity, P, in %, expressing the relationship of the volume of the open pores in the specimen to its exterior volume, is calculated using Equation (1):

(1) $$P=\frac{W-D}{W-S}\times 100$$

The bulk density, BD, in g/cm^3^, is the quotient of its dry weight divided by the exterior volume, including pores, and is calculated by Equation (2):

(2) $$BD=\frac{D}{W-S}$$

The diametral compression test was conducted with a universal electromechanical testing machine (Shimadzu, AGS-X, Japan) with a load cell of 10,000 N [38] and a displacement rate of 0.5 mm/min [39]. The tensile strength, σ_x_, was determined by Equation (3) where F corresponds to the maximum force applied, d, the diameter, and e, the thickness of the cylindric sample:

(3) $$\sigma_{x}=\frac{2\times F}{\pi \times d\times e}$$

**Figure 2:**
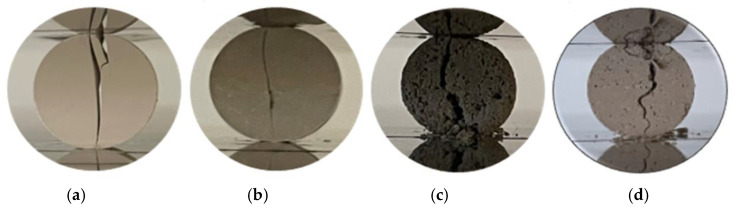
Illustration of a valid diametral compression test: (**a**) 10T and (**b**) 8T2tZ dense samples; (**c**) 10T, and (**d**) 8T2tZ porous samples.

In Figure 2, the illustrations of dense and porous ceramic samples are observed after the fracture occurs during the diametral compression test.

### 2.3. Biological Characterization

The resazurin assay [40] was used to evaluate the cytocompatibility of bioceramic composites in human osteoblasts (hOB). Thus, a cell line of hOB (Cell Applications, Inc., San Diego, CA, USA), t-flasks of 75 cm^3^ cell culture (Orange Scientific, Braine-l’Alleud, Belgium), Dulbecco’s Modified Eagle Medium/Nutrient F-12 (DMEM-F12, Sigma-Aldrich, Sintra, Portugal), sodium bicarbonate (NaHCO_3_, Labchem, Laborspirit, Santo Antão do Tojal, Portugal, CAS: 144-55-8), double deionized water (ultrapure water, obtained using an ultrapure water purification system Milli-Q Advantage A10, filtered at 0.22 μm and 18.2 MΩ cm at 25 °C), bovine fetal serum (FBS, Biochrom AG, Berlin, Germany, CAS: 9014-81-7), trypsin (Sigma-Aldrich, Sintra, Portugal, CAS: 9002-07-7), resazurin (Sigma-Aldrich, Sintra, Portugal, CAS: 62758-13-8), spectrofluorimeter (SpectraMax Gemini EM Molecular Devices, San José, CA, USA), scanning electron microscope (Hitachi, S-3400 N, Tokyo, Japan) and a turbomolecular pumped coater (Quorum Technologies, Q150R ES, Lewes, UK) were used.

Discs similar to those used in the mechanical characterization (Figure 2) of ~13 mm in diameter and 3 mm in thickness were broken into six identical parts, and before starting the biological tests, were sterilized by ultraviolet irradiation (UV) for 1 h [1]. The cells were seeded at a density of 15,000, 10,000, and 2500 cells/well in three 48-well plates for 24 h at 37 °C. Then, the medium was removed, and the cells were incubated with 10% of material in relation to the well area [41] and 300 μL of DMEM-F12 in all wells. After 1, 3, and 7 days of incubation, the medium and the material were removed and the hOB were incubated with 220 μL resazurin 10% (*v*/*v*) [40,42]. Cell viability was determined by measuring resorufin fluorescence at λ_ex_ = 545 nm and λ_em_ = 590 nm [40,42]. The plate corresponding to day 1 of incubation with material contained a density of 15,000 cells/well, day 3, 10,000 cells/well, and day 7 plate, 2500 cells/well. Negative control (K^−^) cells were incubated only with culture medium and positive control (K^+^) cells were incubated with bleach.

The cellular attachment to porous biocomposites was assessed by SEM analysis in BSE 3D mode (Backscattered electrons) with an acceleration voltage of 20 kV. The cells were fixed with 500 μL of glutaraldehyde at 2.5% (*v*/*v*) for 1 h. After, the samples were dehydrated with increasing ethanol concentrations (50, 70, and 99%) for 5 min each, frozen at −80 °C for 1 h and freeze-dried for 3 h. Then, the samples were coated with gold using a turbomolecular pumped coater.

## 3. Results and Discussion

### 3.1. Microstructural Properties

**Figure 3:**
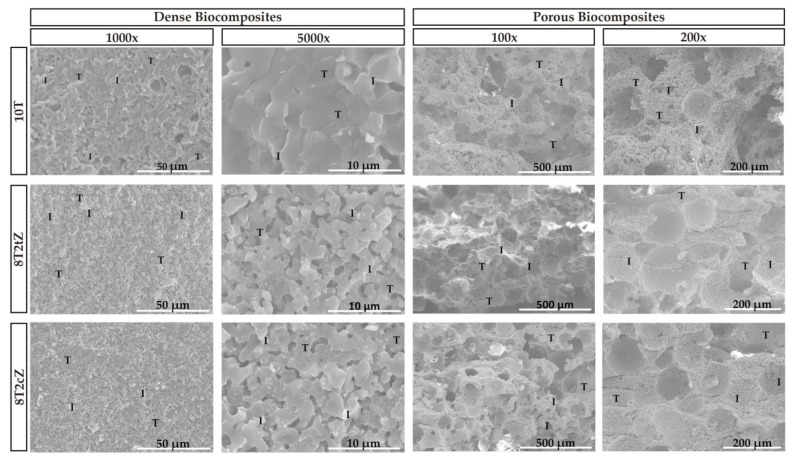
SEM characteristic fracture surfaces of the ceramic biocomposites. Dense biocomposites with a magnification of 1000× and 5000× and porous biocomposites with a magnification of 100× and 200×. Examples of transgranular and intergranular fracture surfaces are illustrated by letters “T” and “I”, respectively.

Representative SEM micrographs of the fracture surfaces of the sintered ceramic composites are shown in Figure 3. In all cases there are mixed fracture surfaces, that is, transgranular (examples illustrated by letter “T” in Figure 3) and intergranular fractures (examples illustrated by letter “I” in Figure 3). Regarding porosity, it is evident that an interconnected porosity network exists even in dense biocomposites. In addition to perfectly spherical pores, that is, pores caused by PMMA spheres, more elongated pores are also observed.

The macropores caused by the PMMA spheres, visible in the image at 200× magnification, present an average size of 100 µm, between 120–150 µm and between 110–140 µm for the 10T, 8T2tZ, and 8T2cZ biocomposites, respectively. In this way, bone growth and cell colonization have good conditions to occur.

**Figure 4:**
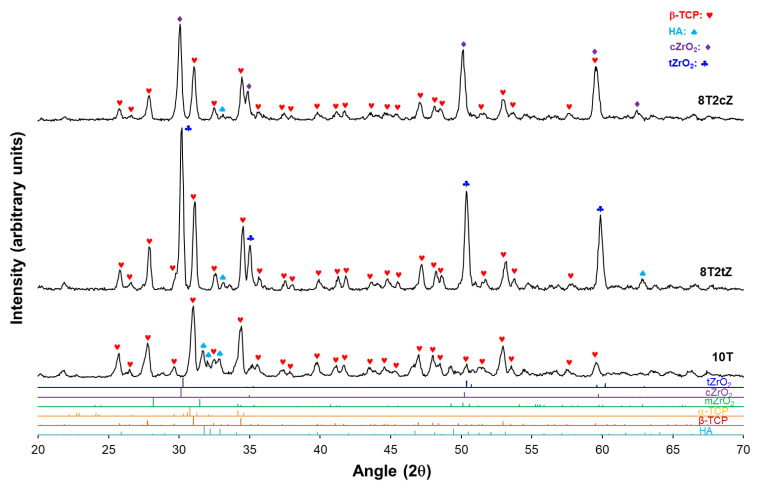
X-ray diffraction spectra of the biocomposites 10T, 8T2tZ, and 8T2cZ and X-ray diffraction spectra of the theoretical cards #37-1484, #50-1089, #49-1642, #09-0432, #09-0169, and #09-0348, corresponding to m-ZrO_2_, t-ZrO_2_ (♣), c-ZrO_2_ (♦), HA (♠), β-TCP (♥), and α-TCP, respectively.

XRD assays were performed to confirm the presence of ZrO_2_ crystalline phases in the dense biocomposites 8T2tZ and 8T2cZ. Figure 4 shows the X-ray diffraction spectra of the analyzed biocomposites. In the 10T biocomposites, in addition to other small, identified peaks, four main peaks of higher intensity were identified for 2θ = 27°, 32°, 34°, and 53° that correspond to β-TCP and are coincident with their theoretical card. The predominant structure is β-TCP (~78%vol according to Table 2). Also, in 10T samples, HA is the second most present phase, and α-TCP, with a very low value, can be neglected. The existence of α-TCP was not expected, because of the doping of TCP with 10% MgO. According to the literature [24], with the addition of Mg^2+^ to TCP, the transformation temperature of β-TCP to α-TCP is expected to increase. This is also promoted by the relative high temperature of sintering. In biocomposites 8T2tZ and 8T2cZ, four main peaks were identified for 2θ = 30.2°, 31.1°, 50.4°, and 59.7° corresponding to t-ZrO_2_, β-TCP, t-ZrO_2_, and also t-ZrO_2_, respectively, for 8T2tZ; in 8T2cZ main peaks for 2θ = 30°, 31.1°, 50.1° and 59.7° were identified, corresponding to c-ZrO_2_, β-TCP, c-ZrO_2_, and also c-ZrO_2_, respectively. For both cases, the peaks coincide with the respective theoretical cards.

The main phases of the biocomposites analyzed were quantified by Rietveld refinement, as shown on Table 2. According to Table 2, the predominant phase is β-TCP, presenting values of 77.60, 71.09, and 77.50%vol for biocomposites 10T, 8T2tZ, and 8T2cZ, respectively. HA was only detected in 8T2tZ and 10T, with values of 3.05 and 21.69%vol, respectively. In biocomposites with 8YSZ, mostly this phase corresponds to ZrO_2_, c-ZrO_2_, whose value is 21.02%vol, and a small percentage of t-ZrO_2_, 1.48%vol, which indicates that during the manufacturing process the c-ZrO_2_ was converted into t-ZrO_2,_ probably due to high temperatures. In the biocomposites with 3YSZ, only t-ZrO_2,_ was quantified, 25.86%vol.

No metal oxides were detected, including MgO, which indicates that during the calcination process, the Ca^2+^ ions were replaced by the metal ions of Mg^2+^, Mn^2+^, Zn^2+^, and Fe^3+^ into sites of the crystalline structure of β-TCP.

In order to understand the presence of several peaks corresponding to HA, the XRD was carried out for sTCP after the calcination process and compared with the XRD after the sintering process (Figure S1). The main peaks correspond, in both spectra, to the TCP beta phase. The presence of TCP alpha crystalline phase peaks is unclear. In the calcined sTCP sample, the peaks for 2θ = 29°, 31.8°, 32.2°, 32.9°, 34.0°, 39.2°, 46.7°, 49.5°, and 63.0° correspond to the hydroxyapatite phase. In the sintered phase at 1300 °C, the number of HA peaks decreases, essentially leaving the relevant peaks at 31.8°, 32.2°, and 32.9°.

The formation of hydroxyapatite in this case most likely occurs through what in the literature is called mechanochemical synthesis [43,44]. These mechanochemical reactions resulted in the formation of a defective phase of calcium-deficient HA, which, when calcined at up to 720 °C, leads to the formation of HA and β-TCP [43]. The principle of this dynamic synthesis during grinding is related to the energy during grinding (the impact that the mill balls have on the powdered grains). In other words, a reaction and an interdiffusion mechanism are promoted between different grains or with an intimate chemical reaction of the molecules between them [44]. In this sense, similar to the present work, after 5 h of mechanical activation, Yeong et al. [43] obtained a 2θ of 31.8° as the most prominent peak, corresponding to the crystalline plane of HA (211).

In fact, the growth of the HA phase, which is directly related to the improvement of biocompatibility and osteoinduction, is important in bone reconstruction [1,18,45].

From the elementary chemical analysis (EDX), Table 3, the presence of the elements Ca, P, Zr, and the remaining added metal ions were verified.

The elements Ca and P come from the chemical formula of TCP, (Ca_3_(PO_4_)_2_), and the Zr from the doping with the two types (tetragonal and cubic) of ZrO_2_ used in the composition of the ceramic biocomposites. The Ca/P ratio, in wt%, of 2.68, 3.53, and 3.37, and in mol% of the 2.07, 2.73, and 2.60 for the ceramic biocomposites 10T, 8T2tZ, and 8T2cZ, respectively is slightly high when compared to the works reported in the literature, namely, Wu et al. [46], which refers to bone minerals with Ca/P ratio between 1.37 and 1.87 mol%. With the addition of the 3YSZ and 8YSZ, this Ca/P ratio, tends to increase, with its highest value being in ceramic biocomposites with 20% of 3YSZ.

**Figure 5:**
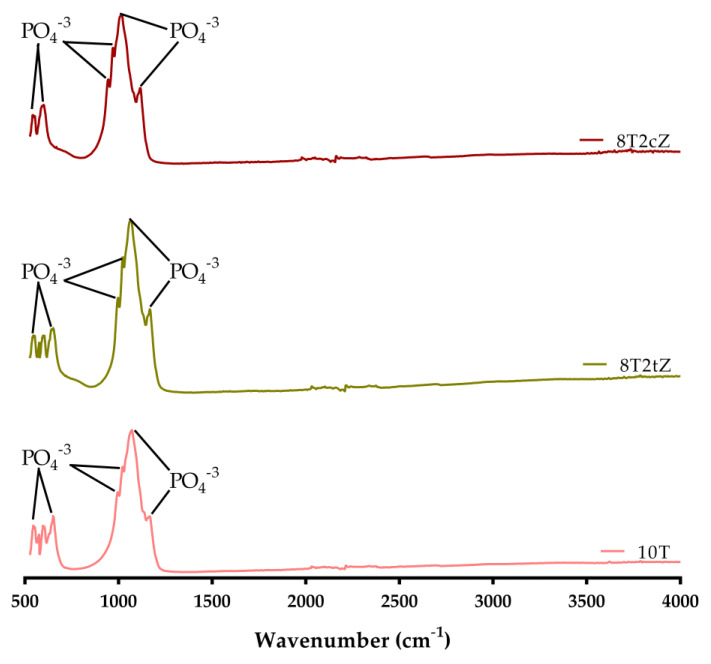
FTIR spectra of different biocomposites 10T, 8T2tZ, and 8T2cZ.

Figure 5 shows the characteristic FTIR spectra of the biocomposites.

From the FTIR analysis, the characteristic covalent bonds of the different ionic groups are observed. The ceramic compound TCP is formed by Ca^2+^ and PO^3−^ ions. The peak at 943.19 cm^−1^ and 972.12 cm^−1^ is related to the presence of pure β-TCP. Thus, the bands between the range 900–1200 cm^−1^ represent the stretching mode of the PO_4_^−3^ group [47,48]. The results show the characteristic peaks of the covalent bonds present in PO_4_^−3^. Hydroxyapatite (HA) has the OH^−^ ion. The characteristic peaks at 630 cm^−1^ and 3571 cm^−1^ were attributed to the stretching mode of the hydroxyl group (OH^−^) [47]; however, these peaks were not clearly detected or were too small compared to others. The band at 465–627 cm^−1^ and the band at 900–1000 cm^−1^ were expected due to Zr-O, which indicates the formation of cubic ZrO_2_ and tetragonal ZrO_2_ crystalline phases, respectively [49,50]. In this sense, it is believed that the peaks with the greater width of the two samples with zirconia (~600 cm^−1^), in comparison with the sharp peaks of the 10T composition, and the more pronounced drop (~820 cm^−1^), are due to the presence of Zr-O bonds, thus being almost undetectable due to their low content and the presence of high PO_4_^3−^ peaks in this region.

### 3.2. Mechanical Properties

The apparent porosity of the 10T biocomposite was 12.9%. The porosity increased to 24.6% and 21.9% for the dense biocomposites reinforced with 3YSZ, i.e., 9T1tZ and 8T2tZ, respectively. The similar samples manufactured with PMMA show higher porosity, namely, 26.2%, 29.0%, and 29.3% for 10T, 9T1tZ, and 8T2tZ, respectively. The dense biocomposites of 9T1cZ and 8T2cZ showed lower apparent porosity with reinforcement of 10 wt% of 8YSZ (14.9%) and similar porosity for 20 wt% (20.7%). The samples manufactured with PMMA present similar porosity of 28.9% and 27.6%, for 10 wt% and 20 wt%, respectively. In both manufacturing conditions, the addition of 3YSZ or 8YSZ increased the apparent porosity in relation to the standard, 10T, see Figure 2, Figure 3 and Figure S2.

The bulk density of the sTCP doped with metal ions (reference material, 10T) presented a value of 2.80 g/cm^3^. The density increased for 3.19 g/cm^3^ and 3.36 g/cm^3^ and for 3.18 g/cm^3^ and 3.35 g/cm^3^ for the dense biocomposites reinforced with 3YSZ and 8YSZ, respectively. The samples manufactured with PMMA, with 10 wt% and 20 wt% of 3YSZ, present a density of 2.30 g/cm^3^ and 2.62 g/cm^3^, respectively. While the porous samples reinforced with 10 wt% and 20 wt% of 8YSZ, show 2.73 g/cm^3^ and 2.60 g/cm^3^, respectively. The addition of 3YSZ and 8YSZ in dense biocomposites increased the density related to initial doped sTCP. However, in the samples manufactured with PMMA the density decreased or presented similar values (Figure 3 and Figure S3).

These results are in agreement with the literature [1,18,31,45]. There, it was proven that, with the addition of 10 and 20% ZrO_2_, the apparent porosity increased while the bulk density decreased, and this is what happened with the porous biocomposites analyzed in this work. With the addition of the 3YSZ and 8YSZ, porosity and bulk density increased, which can be explained by the effect of the sintering temperature (1300 °C). Considering the particle size distribution of the mixture up to 10 µm, with both zirconias up to 2 µm, the sintering conditions by pressure-less, unassisted sintering where, in general, densification is accompanied by (an undesirable) grain coarsening, the success of avoiding the grain growth is related to the control of the competition between densification and grain growth. That is extremely difficult because the driving forces for both are proportional to the reciprocal grain size and hence comparable in magnitude [51]. Thus, for the composition, particle size, and single step sintering in air the temperature used is not sufficient to promote the grain boundary atomic migration of zirconia. In these conditions, only the approximation of zirconia particles promotes the formation of zirconia agglomerates and partial grain growing with poor densification and superior porosity [52]. Nonetheless, the addition of ions like Mg^2+^ promotes the densification of materials [18,45]. Another explanation for the apparent porosity results in the use of the biocomposites manufactured with PMMA (40 wt%). Lee et al. [31] showed that, with different percentages of PMMA, starting with 40%, the porosity tends to increase gradually, and even with 40% the value is already quite considerable.

The diametrical compression tests revealed that the dense biocomposites with 3YSZ and 8YSZ presented mechanical strength values between 12.85 and 16.40 MPa and between 13.36 and 20.74 MPa, respectively (Table 4).

These results revealed that, in comparison with sTCP doped (13.4 MPa), the dense biocomposites with 20 wt% of 3YSZ and 8YSZ are higher. The samples 8T2cZ, are those that have higher mechanical strength, with a value of 20.7 MPa (55% higher than reference doped sTCP). As expected in induced porous biocomposites, the mechanical strength values were greatly decreased. From the initial 1.28 MPa (TCP doped) the addition of the 20 wt% of ZrO_2_ decreases to 0.1 and 0.14 MPa for 3YSZ and 8YSZ, respectively (Figure S4).

The addition of ZrO_2_ to the ions doped with sTCP creates a ceramic microstructure in which the sTCP base matrix is reinforced with micro- and nanoparticles of zirconia (3YSZ and 8YSZ) which is due to its superior mechanical resistance (strength, toughness, and hardness). Furthermore, it is evident that the superior mechanical properties of the cubic phase (8YSZ) contribute more effectively to the increase in resistance than the tetragonal phase (3YSZ) [53].

Similar to previous studies, biocomposites with reinforcement of ZrO_2_ have higher mechanical strength than “pure” biocomposites, i.e., biocomposites without the presence of ZrO_2_ [1,54,55]. In this study the most resistant biocomposites have 20% of 3YSZ or 8YSZ. On the other hand, these results can also be explained by the addition of Mg^2+^, that replaces the Ca^2+^ ions in its sites (doping effect), which have already been shown to increase the mechanical strength of the biocomposites [45]. Regarding biocomposites manufactured with PMMA, low mechanical strength was already reported by Lee et al. [31].

### 3.3. Biological Properties

**Figure 6:**
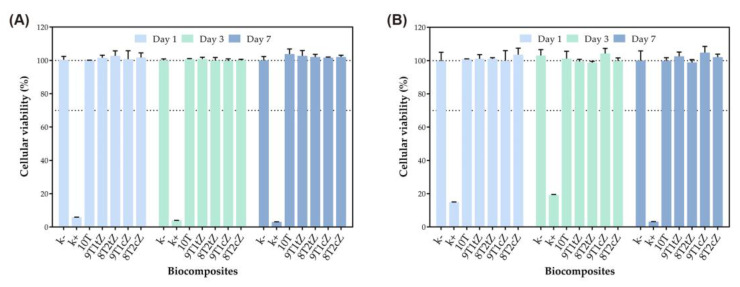
Evaluation of the cytocompatibility of biocomposites: (**A**) dense samples reinforced with 3YSZ and 8YSZ; (**B**) porous samples reinforced with 3YSZ and 8YSZ.

The biocompatibility of dense ceramic biocomposites, both with and without porosity induced by PMMA reinforcement with 3YSZ and 8YSZ, was evaluated using a resazurin assay, with the corresponding results displayed in Figure 6. hOB were chosen as the cell model due to their pivotal role in bone matrix production and remodeling [56], a crucial aspect of the osseointegration process. Notably, the data obtained from the resazurin assay, as shown in Figure 6A,B, demonstrated that even after 7 days of incubation, the hOB cells remained highly metabolically active when in contact with both dense and porous biocomposites.

In fact, their cell viability consistently exceeded the 70% threshold, indicating strong biocompatibility. It is worth noting that the porous biocomposites were made from PMMA, a structure porosity that, according to the literature [57], could potentially hinder biocompatibility and bioactivity. However, the results of this study are consistent with previous research investigating the in vitro cytotoxicity of biocomposites containing β-TCP and ZrO_2_ [1,45,58]. Also, as in other reported studies [57,58], the use of PMMA during manufacturing did not leave residues that could harm the cytotoxicity results.

As in previous studies [45], no relevant differences were detected in cell viability and proliferation between the pure TCP composition and the TCP compositions doped with combinations of the four metal ions, while the bioceramic composites of TCP with zirconia did not show high cell viability. This cell viability can be attributed to the range of molar concentrations of ions incorporated into the crystalline structure of β-TCP, which closely mimics the composition of natural human bone [14,45,59].

**Figure 7:**
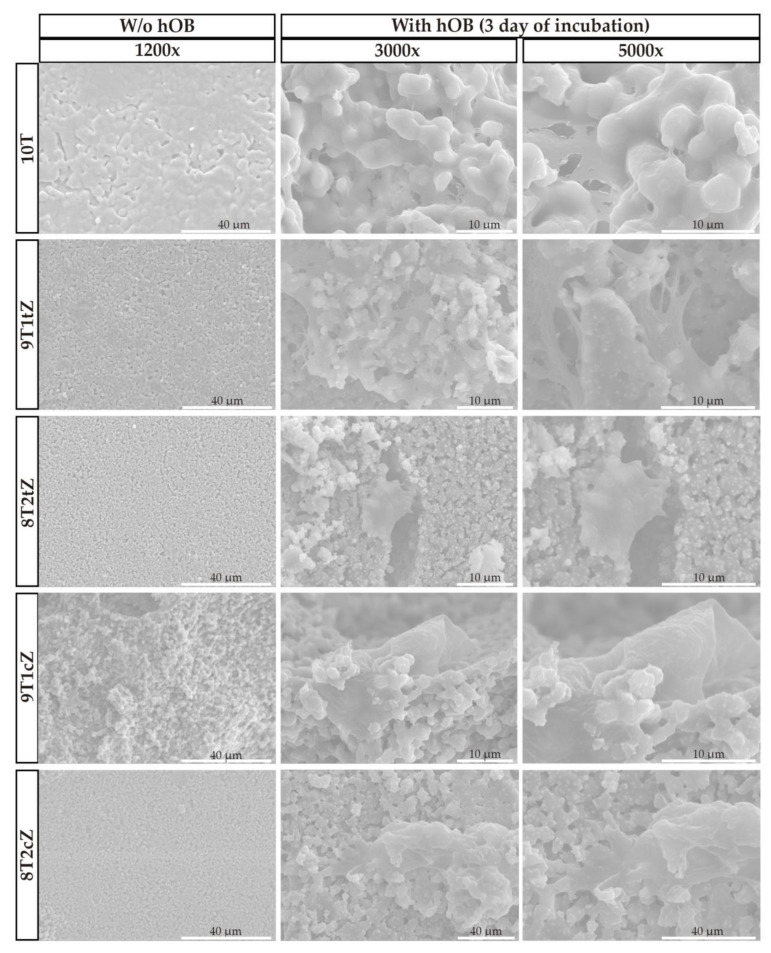
SEM images of cellular attachment to porous biocomposites after 3 days and without cellular attachment, with magnifications of 3000×, 5000× and 1200×, respectively.

SEM was used to visualize cell attachment and growth on the PMMA-induced porous biocomposites. Figure 7 shows the results at 3000× and 5000× magnification after 3 days of incubation. In addition, SEM images of the biocomposites without hOB cells, magnified to 1200×, are included for comparison.

These images clearly show that hOB cells not only adhered to the surface but also infiltrated the interior of the biocomposites over time. This observation is consistent with previous research using β-TCP [60]. These results may be associated with the porosity present in the materials, whose macropores have an ideal average size between 100–200 μm for cell growth, and according to the literature [61,62,63] this directly influences cell proliferation, promoting cell adhesion and growth.

## 4. Conclusions

Ceramic biocomposites with different percentage of metal ion doped sTCP, 3YSZ, and 8YSZ, in dense form and with porosity induced by PMMA, were manufactured. The ceramic microstructure and the composition obtained have a good similarity with human bone biomimicry. The effect of doping with 15 mol% metal ions of Mg^2+^, Zn^2+^, Mn^2+^, and Fe^3+^ and simultaneously strengthening with two ZrO_2_ (tetragonal and cubic crystalline phases) was assessed through the physical, mechanical, microstructural, and biological properties.

XRD, EDX, and FTIR analyses show that metal ions are present in the composition and replace the calcium in the crystal structure of the biocomposites. The β-TCP crystalline phase is predominant; however, hydroxyapatite is also present in the reference material (sTCP), most likely due to mechanochemical synthesis during the mixing/grinding process. As expected, the tetragonal and cubic phases are also present in the biocomposites reinforced by the respective ZrO_2_. The images of the fracture surfaces show the porosity, interconnectivity, and fracture modes, while in the materials with induced porosity, voids generated by the PMMA spheres of approximately 100 microns are observed.

Moreover, from the analysis of the results, the following summary can be given:

(i) The addition of 20 wt% of 3YSZ increases the mechanical strength to 16.4 MPa, 22% higher than sTCP; and 20 wt% of 8YSZ increases the strength to 20.7 MPa, 55% highest than reference doped sTCP. In this way, as expected, cubic zirconia promotes greater resistance than tetragonal zirconia.
(ii) The apparent porosity increases 65%, from 12.6 to 24.6% with the addition of 10 wt% of 3YSZ and 60%, to 20.6% with the addition of 20 wt% of 8YSZ. This is justified by the sintering conditions of pressure-less, unassisted sintering, where the temperature is not sufficient to promote atomic migration along the zirconia grain boundary, promoting the formation of zirconia agglomerates and partial grain growing with poor densification.
(iii) The use of PMMA increases the apparent porosity for all ceramic biocomposites; however, this effect is more visible for sTCP, where the value obtained of 26.2%, matches an increase of two times. In this case the mechanical resistance is too low for structural applications.
(iv) The resazurin assay revealed that the two types of biocomposites produced did not affect the viability of hOB, presenting a cellular viability, in most cases, of 100%. These results highlight the ideal properties of these biocomposites as bone substitutes, especially the dense ones doped with 8YSZ, which showed a more constant behavior for the analyzed properties.

## Figures and Tables

**Table 1:** Nomenclature and composition of the materials studied in molar fraction (x_i_), volumetric fraction (v_i_) and mass fraction (w_i_).

Materials	Molar Fraction x_i_			Volumetric Fraction v_i_			Mass Fraction w_i_		
	sTCP	3YSZ	8YSZ	sTCP	3YSZ	8YSZ	sTCP	3YSZ	8YSZ
10T	1	0	0	1	0	0	1	0	0
9T1tZ	0.7814	0.2186	0	0.9473	0.0527	0	0.9	0.1	0
8T2tZ	0.6138	0.3862	0	0.8888	0.1112	0	0.8	0.2	0
9T1cZ	0.7814	0	0.2186	0.9468	0	0.0532	0.9	0	0.1
8T2cZ	0.6138	0	0.3862	0.8877	0	0.1123	0.8	0	0.2

**Table 2:** Quantitative analysis (%vol) of XRD for bioceramic composites 10T, 8T2tZ, and 8T2cZ, by Rietveld refinement.

Phases (%vol)	10T	8T2tZ	8T2cZ
β-TCP	77.60 ± 0.00	71.09 ± 1.00	77.50 ± 0.00
HA	21.9 ± 0.00	3.05 ± 0.02	-
α-TCP	0.72 ± 0.00	-	-
t-ZrO_2_	-	25.86 ± 0.54	1.48 ± 0.00
c-ZrO_2_	-	-	21.02 ± 0.00

**Table 3:** Chemistry analysis (EDX) of the ceramic biocomposites 10T, 8T2tZ, and 8T2cZ. The error is the standard deviation (sd).

Element	10T			8T2tZ			8T2cZ		
	wt%	mol%	sd wt%	wt%	mol%	sd wt%	wt%	mol%	sd wt%
Ca	46.7	30.02	2.2	36.8	24.61	2.01	37.93	28.65	2.03
P	17.43	14.5	1.12	10.42	9.02	0.79	11.24	10.95	0.84
O	33.51	53.97	9.09	36.33	60.87	9.76	27.59	52.04	7.85
Mg	0.78	0.82	0.15	0.65	0.72	0.14	0.62	0.78	0.13
Mn	0.47	0.22	0.11	0.35	0.17	0.09	0.25	0.14	0.09
Fe	0.48	0.22	0.11	0.14	0.07	0.08	0.31	0.17	0.09
Zn	0.64	0.25	0.13	0.37	0.15	0.11	0.55	0.25	0.12
Zr	-	-	-	14.95	4.39	1.1	21.5	7.11	1.52

**Table 4:** Diametral compression for dense and porous bioceramic composites.

sTCP	sTCP + t-ZrO_2_		sTCP + c-ZrO_2_	
10T	9T1tZ	8T2tZ	9T1cZ	8T2cZ
Dense biocomposites				
13.36 ± 1.6	12.85 ± 1.6	16.40 ± 1.4	15.22 ± 1.5	20.74 ± 1.6
Porous biocomposites				
1.28 ± 0.3	0.16 ± 0.1	0.10 ± 0.0	0.20 ± 0.0	0.14 ± 0.1

## Data Availability

The authors declare that the data supporting the findings of this study are available within the article.

## Supplementary Materials

The following supporting information can be downloaded at: https://www.mdpi.com/article/10.3390/biomimetics8080599/s1, Figure S1: X-ray diffraction spectra of the biocomposites 10T (sTCP) after calcination and after sintering and X-ray diffraction spectra of the theoretical cards #09-0432, #09-0169, and #09-0348, corresponding to HA, β-TCP and α-TCP, respectively; Figure S2: Apparent porosity of the composites: (a) dense reinforced with 3YSZ; (b) manufactured with PMMA and reinforced with 3YSZ; (c) dense reinforced with 8YSZ; (d) manufactured with PMMA and reinforced with 8YSZ; Figure S3: Bulk density of the composites: (a) dense reinforced with 3YSZ; (b) manufactured with PMMA and reinforced with 3YSZ; (c) dense reinforced with 8YSZ; (d) manufactured with PMMA and reinforced with 8YSZ; Figure S4: Mechanical strength of the composites: (a) dense reinforced with 3YSZ; (b) manufactured with PMMA and reinforced with 3YSZ; (c) dense reinforced with 8YSZ; (d) manufactured with PMMA and reinforced with 8YSZ.
