# Supplementary material for: TCP Doped with Metal Ions Reinforced with Tetragonal and Cubic Zirconia
Source: Biomimetics (Basel). 2023 Dec 12;8(8):599. doi: 10.3390/biomimetics8080599 (PMC10742230; doi:10.3390/biomimetics8080599)
Supplement: Supplementary file 1 [file biomimetics-08-00599-s001.zip › biomimetics-2695660-supplementary.pdf]

Supplementary Materials

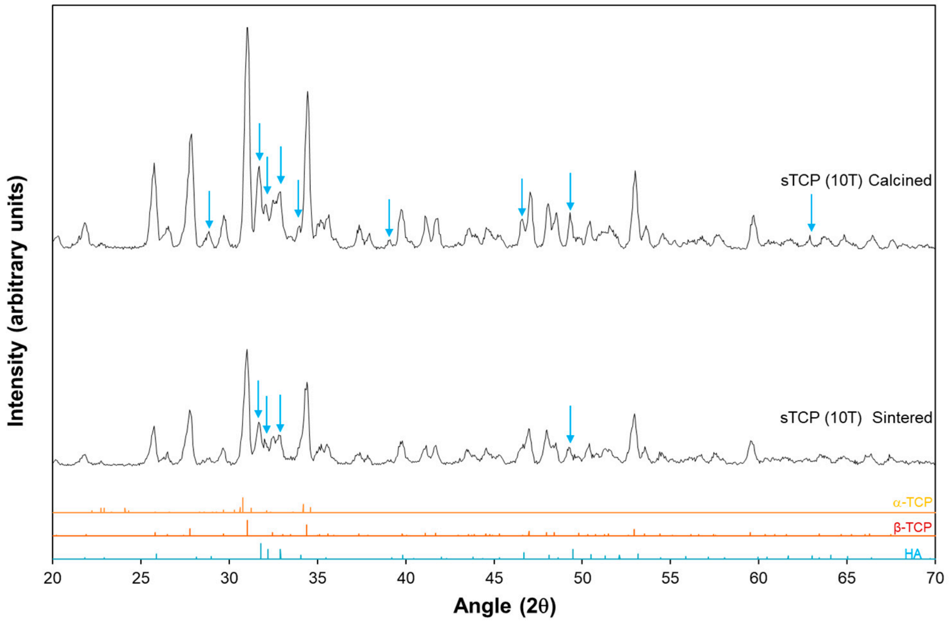

Figure S1. X-ray diffraction spectra of the biocomposites 10T (sTCP) after calcination and after sintering and X-ray diffraction spectra of the theoretical cards #09-0432, #09-0169, and #09-0348, corresponding to HA, β-TCP, and α-TCP, respectively.

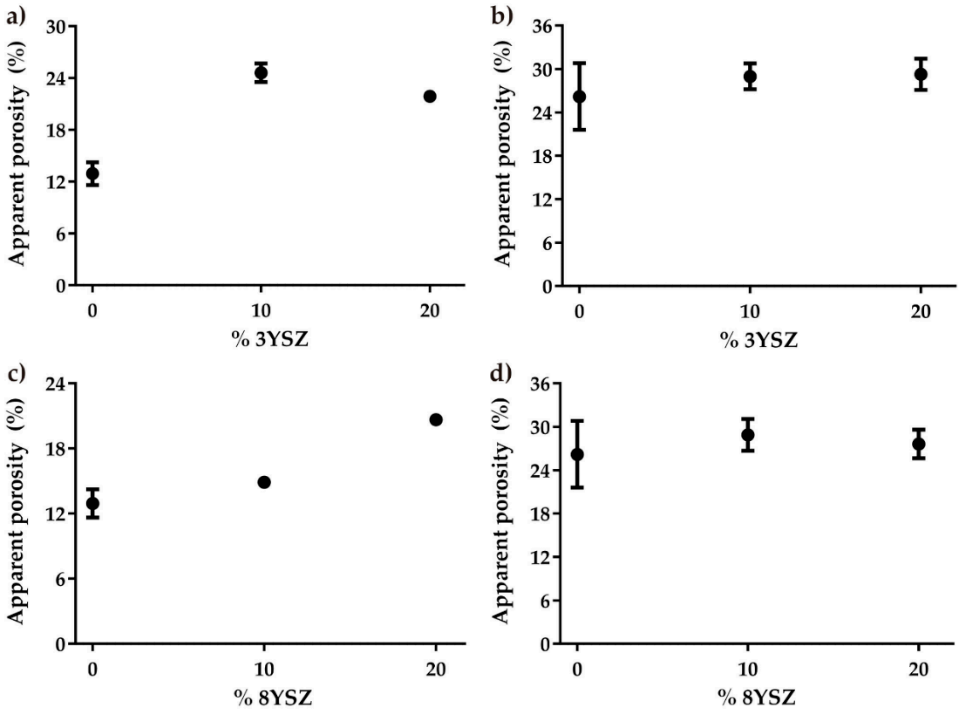

Figure S2. Apparent porosity of the composites: a) dense reinforced with 3YSZ; b) manufactured with PMMA and reinforced with 3YSZ; c) dense reinforced with 8YSZ; d) manufactured with PMMA and reinforced with 8YSZ.

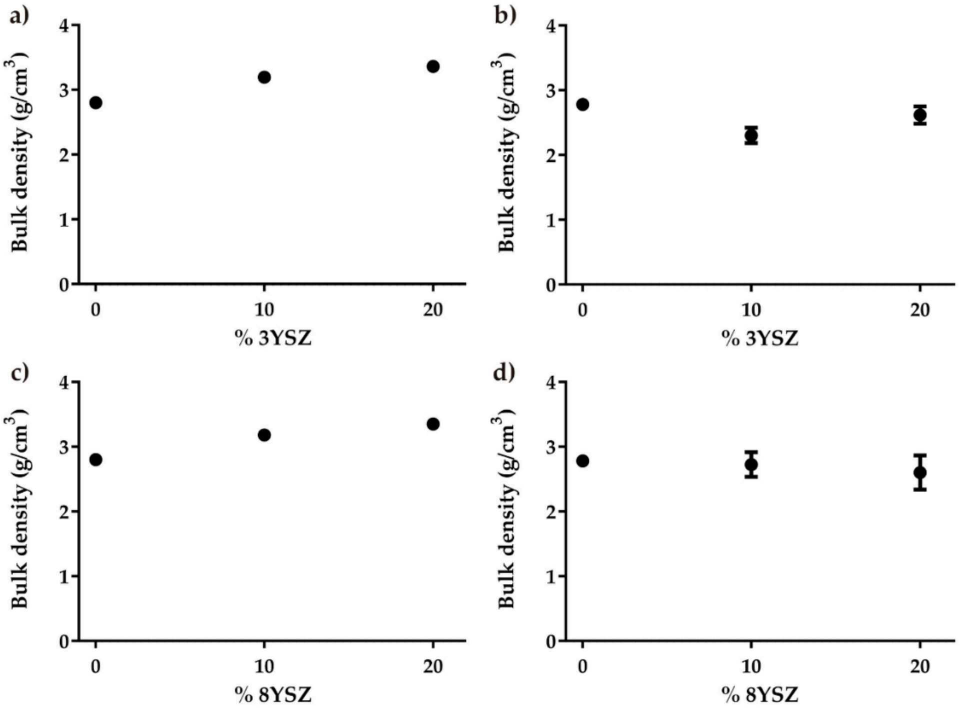

Figure S3. Bulk density of the composites: a) dense reinforced with 3YSZ; b) manufactured with PMMA and reinforced with 3YSZ; c) dense reinforced with 8YSZ; d) manufactured with PMMA and reinforced with 8YSZ.

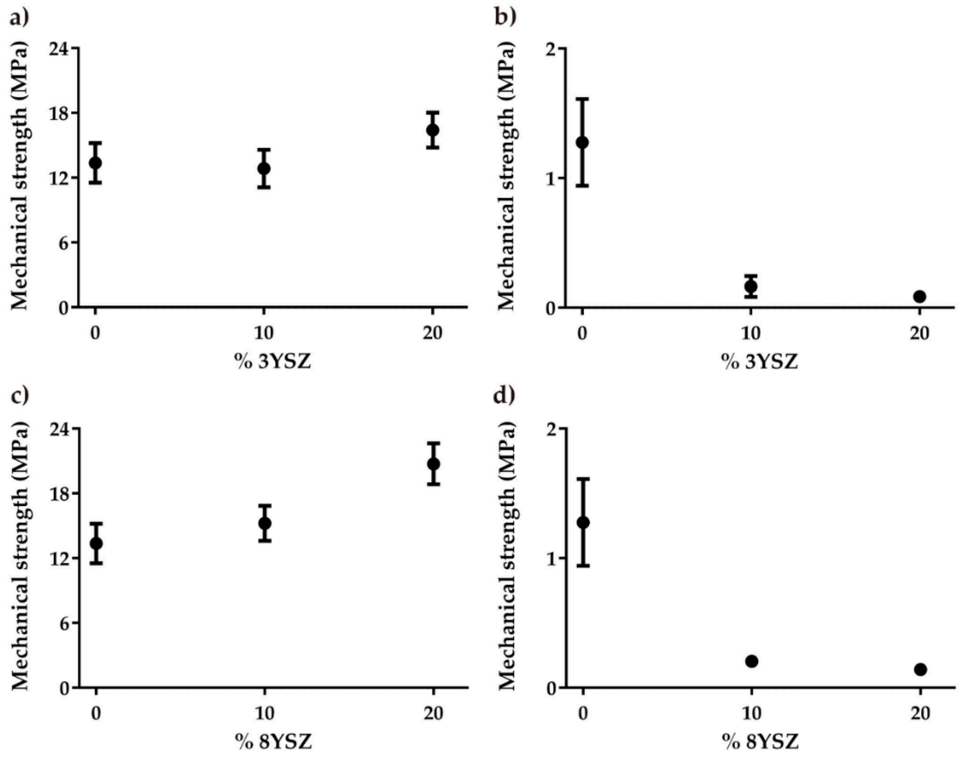

Figure S4. Mechanical strength of the composites: a) dense reinforced with 3YSZ; b) manufactured with PMMA and reinforced with 3YSZ; c) dense reinforced with 8YSZ; d) manufactured with PMMA and reinforced with 8YSZ.
